# The Initiation of Laparoscopic Gastric Surgery in an Academic Hospital in Greece: Early Results

**DOI:** 10.7759/cureus.112507

**Published:** 2026-07-12

**Authors:** Vasilios Zoubos, Nikolaos S Georgopoulos, Theodoros Sidiropoulos, Vasilios Tsaousis, Nikolaos Arkadopoulos, Ioannis Hatzaras

**Affiliations:** 1 4th Department of Surgery, Attikon University Hospital, National and Kapodistrian University of Athens, Athens, GRC

**Keywords:** gastric cancer, laparoscopic gastrectomy, lymphadenectomy, minimally invasive surgery, perioperative outcomes

## Abstract

Background and objective

Recent studies have demonstrated that minimally invasive techniques are a viable alternative to open surgery for the treatment of gastric cancer (GC), providing both intraoperative and postoperative benefits for patients. In addition, preliminary data suggest that laparoscopic gastrectomy (LG) may have morbidity, mortality, and oncologic outcomes comparable to those of the traditional open approach for treating GC. This study aimed to review our institution's experience with totally laparoscopic gastrectomy for the treatment of early- and advanced-stage GC.

Methods

A retrospective study was conducted to examine the short-term outcomes of LG performed at Attikon University Hospital in Athens between June 2022 and November 2024. We reviewed postoperative complications, surgical margins, the number of resected lymph nodes, estimated blood loss, length of hospital stay, and recurrence rates.

Results

Fourteen patients with gastric adenocarcinoma were included in the study. The mean operative time was four hours, and blood loss was minimal. The median length of hospital stay was six days. The mean number of harvested lymph nodes was 16. Early postoperative complications occurred in two patients and included delayed gastric emptying and pulmonary infection. There were no postoperative deaths.

Conclusions

Totally laparoscopic gastrectomy represents a feasible treatment option for gastric malignancy. Our early institutional experience demonstrates the technical feasibility of this approach, with favorable perioperative outcomes and encouraging short-term survival. While the benefits of the laparoscopic approach are well recognized, larger prospective studies with long-term follow-up are required to confirm its oncologic equivalence or superiority over open gastrectomy (OG) in patients with advanced disease.

## Introduction

Gastric cancer (GC) remains one of the most common causes of cancer‑related mortality worldwide despite a gradual decline in incidence in many regions. According to recent global estimates, there are over one million new cases of GC annually [1-3], ranking it fifth among all cancer types. Moreover, data from 2020 indicate that approximately 770,000 deaths were attributable to GC globally, ranking it fourth among all cancer types after lung, colorectal, and liver cancer [4,5]. The projected annual burden of gastric cancer is expected to reach approximately 1.8 million new cases and around 1.3 million deaths by 2040 [6].

The disease burden in Europe is heterogeneous, with Southern European and Mediterranean countries continuing to record substantial GC incidence [7]. In the European Union, stomach cancer remains a significant contributor to cancer morbidity and mortality [7]. In Greece, although national data are limited, GC remains an important cause of cancer mortality [8]. The lack of widespread screening programs, combined with the typically late presentation of the disease due to nonspecific early symptoms, continues to hinder early detection, a pattern consistent with European and global observations.

Historically, the mainstay of curative treatment for GC has been open subtotal or total gastrectomy (TG) with lymphadenectomy, combined with conventional perioperative care. Over time, refinements in surgical technique, anesthesia, and postoperative management have gradually reduced morbidity and mortality [9,10]. Nevertheless, open surgery remains associated with significant surgical stress, considerable tissue trauma, prolonged recovery, greater postoperative pain, and a higher risk of complications, particularly in older patients and those with comorbidities [11]. This has fueled the continued adoption of minimally invasive alternatives over the past three decades.

Laparoscopic gastrectomy (LG), which was initially introduced for early-stage GC in Asia, gained traction in the 1990s with improvements in instrumentation and growing surgical expertise [12]. Numerous randomized controlled trials and high-quality cohort studies, primarily from East Asia, have demonstrated that laparoscopic distal gastrectomy is associated with reduced intraoperative blood loss, shorter hospital stays, faster recovery, and comparable long-term oncologic outcomes compared with open surgery for early-stage disease [13-15]. Interest has also grown in total LG and its use in Western populations [16], where patients typically have higher body mass indices and present with more advanced disease.

In Western populations (Europe and North America), adoption of LG has been slower, influenced by lower incidence and consequently lower surgical case volumes, differences in surgical training, and initial skepticism regarding oncologic adequacy in advanced disease [17]. However, accumulating evidence supports the conclusion that, in experienced hands, LG yields short-term benefits such as less blood loss, shorter hospital stay, and faster recovery, while providing comparable long-term outcomes to open gastrectomy (OG) [17,18]. Specifically, a meta-analysis of Western centers including 24,098 patients that compared LG with OG reported significantly lower blood loss, shorter hospital stays, reduced analgesic requirement, and lower mortality with LG, without significant differences in lymph node yield or major complications [17,19]. These data suggest that LG is an effective, safe, and oncologically sound approach in Western practice when performed under appropriate conditions.

In this context, the Fourth Department of Surgery at Attikon University Hospital in Athens, Greece, launched a program of totally laparoscopic gastrectomies for both early- and advanced-stage gastric adenocarcinoma in mid-2022. As an academic center committed to innovation and surgical training, the hospital aimed to assess not only the technical feasibility of this approach but also its safety and short-term outcomes in a Western European setting. This paper reports our early experience, highlighting operative metrics, postoperative recovery, and pathological findings.

By sharing these results, we aim to contribute to the growing evidence supporting minimally invasive gastric surgery in non-Asian populations and provide a reference for other Greek and European centers considering the adoption of this technique. The primary objective of this study was to describe our initial institutional experience with totally laparoscopic gastrectomy and to evaluate the feasibility and early perioperative outcomes associated with this approach during its initial implementation at our institution. Secondary objectives included assessing postoperative morbidity, mortality, oncologic adequacy, and short-term survival outcomes.

This article was previously presented as a poster at the 16th International Gastric Cancer Congress, held in Amsterdam, the Netherlands, from May 7 to May 10, 2025.

## Materials and methods

Study design and setting

We conducted a retrospective observational study at Attikon University Hospital, a major tertiary care academic center in Athens, Greece. The hospital provides comprehensive oncologic care and serves as a referral center for gastric malignancies from across the region. The study was approved by the institutional ethics committee, and all patients provided informed consent for the use of their data for research purposes.

Patient selection

All consecutive patients undergoing LG for histologically confirmed gastric adenocarcinoma between June 2022 and November 2024 were included. Patients undergoing palliative or emergency procedures were excluded. Data were collected from electronic medical records, operative reports, and pathology files.

Sample size considerations

Given the retrospective design of the study, all consecutive patients who met the predefined inclusion criteria and underwent LG during the study period were included in the analysis. The final study cohort therefore reflected the total number of eligible cases treated at our institution during the study period.

Surgical technique

All procedures were performed by a team of surgeons trained in advanced laparoscopic and surgical oncology techniques. Depending on tumor location and stage, patients underwent either total or distal gastrectomy with D1+ or D2 lymphadenectomy, following European Society for Medical Oncology (ESMO) guidelines [20]. Surgeries were performed under general anesthesia with the patient in a reverse Trendelenburg position, typically using five trocars. Key steps included full mobilization of the stomach, division of the duodenum or esophagus as appropriate, en bloc lymph node dissection of the hepatogastric ligament with skeletonization of celiac artery branches, and intracorporeal reconstruction via Roux-en-Y or Billroth II anastomosis. Specimens were retrieved through an enlarged trocar site using a wound protector. Perioperative care adhered to standardized Enhanced Recovery After Surgery (ERAS) protocols [21].

Outcome measures

Primary outcomes included intraoperative metrics (operative time, estimated blood loss), postoperative recovery (length of hospital stay, complications within 30 days), and pathologic findings (margin status, number of lymph nodes retrieved). Complications were classified according to the Clavien-Dindo system [22]. Secondary outcomes included early recurrence within the follow-up period. Demographic data (age, sex, BMI, comorbidities, tumor stage) were also recorded.

Statistical analysis

Given the small sample size, only descriptive statistics were applied. Continuous variables are reported as means or medians with ranges, while categorical variables are expressed as counts and percentages. All analyses were conducted using STATA statistical software, Release 18 (StataCorp LLC, College Station, TX).

## Results

Patient characteristics

Between June 2022 and November 2024, 14 patients met the inclusion criteria and were included in the study. Baseline demographic and clinicopathologic characteristics are summarized in Table 1. The mean age was 64 years (range: 48-78 years), with nine males and five females. The median BMI was 27 kg/m². Comorbidities included hypertension in seven patients (50%), diabetes mellitus in four patients (30%), and chronic obstructive pulmonary disease in one patient (10%). Four patients had early-stage (stage I) disease, while 10 had locally advanced (stage II-III) tumors. A total of seven patients underwent TG, while another seven patients underwent subtotal gastrectomy.

**Table 1:** Demographic and clinical characteristics of the patients Data are presented as n (%) for categorical variables and median (range) or mean (range) for continuous variables, as appropriate. Age is reported as mean (range), whereas body mass index is reported as median BMI: body mass index; COPD: chronic obstructive pulmonary disease

Characteristic	Value
Number of patients	14
Age, years, mean (range)	64 (48–78)
Male sex, n (%)	9 (64.3)
Female sex, n (%)	5 (35.7)
BMI, kg/m², median (range)	27 (21–31.5)
Hypertension, n (%)	7 (50.0)
Diabetes mellitus, n (%)	4 (28.6)
COPD, n (%)	1 (7.1)
Stage I disease, n (%)	4 (28.6)
Stage II–III disease, n (%)	10 (71.4)
Total gastrectomy, n (%)	7 (50.0)
Subtotal gastrectomy, n (%)	7 (50.0)

Intraoperative outcomes

The mean operative time was approximately four hours (range: 3.5-5.5 hours). Estimated blood loss was minimal in all cases, with a mean of 80 mL (range: 40-150 mL). Two of the procedures required conversion to open surgery. In both cases, conversion was performed to ensure safe completion of the procedure while maintaining oncologic principles. There were no intraoperative complications such as major bleeding or organ injury, and there were no intraoperative deaths.

Postoperative recovery

The median length of hospital stay was six days (range: five to nine days). Most patients resumed oral intake by postoperative day three. Early postoperative complications occurred in two patients (14.3%): one experienced delayed gastric emptying requiring nasogastric decompression for several days, and another developed a lung infection, classified as Clavien-Dindo grade II, managed with intravenous antibiotics. There were no anastomotic leaks, hemorrhages, or reoperations. No deaths occurred during the perioperative period. Intraoperative and postoperative outcomes are presented in Table 2.

**Table 2:** Intraoperative and postoperative outcomes Data are presented as n (%) for categorical variables and median (range) or mean (range) for continuous variables, as appropriate. Operative time and estimated blood loss are reported as mean (range), whereas length of hospital stay is reported as median (range). Resumption of oral intake is expressed as the median postoperative day. Perioperative mortality was defined as death occurring during the index hospitalization or within 30 days after surgery

Outcome	Value
Operative time, hours, mean (range)	4.0 (3.5–5.5)
Estimated blood loss, mL, mean (range)	80 (40–150)
Conversion to open surgery, n (%)	2 (14.3)
Intraoperative complications, n (%)	0
Intraoperative mortality, n (%)	0
Length of hospital stay, days, median (range)	6 (5–9)
Overall postoperative complications, n (%)	2 (14.3)
Delayed gastric emptying, n (%)	1 (7.1)
Pulmonary infection, n (%)	1 (7.1)
Anastomotic leak, n (%)	0
Postoperative hemorrhage, n (%)	0
Reoperation, n (%)	0
Perioperative mortality, n (%)	0

Pathologic and short-term oncologic outcomes

Pathologic and short-term oncologic outcomes are summarized in Table 3. All patients achieved an R0 resection, with negative microscopic margins in all specimens. The mean number of harvested lymph nodes was 16 (range: 14-20). Among the 10 patients with stage II-III disease, six (60.0%) received adjuvant chemotherapy. After a median follow-up of 10 months (range: 2-18 months), no local recurrences were observed. One patient with stage III disease developed liver metastases 12 months after surgery. At the time of last follow-up, 13 patients (92.9%) were alive without evidence of disease, while one patient was alive with recurrent metastatic disease. No deaths were recorded during follow-up.

**Table 3:** Pathologic and oncologic outcomes Data are presented as n (%) for categorical variables and median (range) or mean (range) for continuous variables, as appropriate. Harvested lymph nodes are reported as mean (range) and follow-up as median (range). Percentages for adjuvant chemotherapy are calculated among patients with stage II–III disease. R0 resection indicates microscopically margin-negative resection

Outcome	Value
Negative resection margins (R0), n (%)	14 (100)
Harvested lymph nodes, mean (range)	16 (14–20)
Patients with advanced disease (stage II–III), n	10
Adjuvant chemotherapy among stage II–III patients, n (%)	6 (60.0)
Median follow-up, months (range)	10 (2–18)
Local recurrence, n (%)	0
Distant recurrence (liver metastasis), n (%)	1 (7.1)
Alive without evidence of disease, n (%)	13 (92.9)
Overall survival, n (%)	14 (100)

## Discussion

Our initial experience with totally laparoscopic gastrectomy in an academic Greek hospital demonstrates that the procedure is feasible and safe. Operative times were within the expected range for a program in its early stages, blood loss was minimal, and complication rates were low. Importantly, the mean lymph node yield of 16 compares favorably with published benchmarks and suggests the oncologic adequacy of the dissections performed.

The benefits of LG have been well documented in East Asian studies, particularly for distal gastrectomy performed for early cancer. For instance, the landmark KLASS-01 trial in Korea demonstrated non-inferior long-term survival and fewer short-term complications compared with open surgery [13]. More recently, trials such as CLASS-01 in China and multicenter European cohorts have extended these findings to TG and more advanced disease [14]. Our results are consistent with the range of outcomes reported in these studies, with low morbidity and acceptable lymph node harvests, despite our patients having higher BMIs and a higher proportion of advanced cases than those in typical Asian cohorts.

The adoption of LG in Western institutions has accelerated over the last decade, supported by a growing body of high-quality evidence demonstrating improved short-term outcomes and comparable long-term oncologic safety relative to open surgery. A large meta-analysis from Western cohorts of 24,098 patients showed that LG significantly reduces blood loss, accelerates postoperative recovery, shortens hospital stay, and lowers mortality, while maintaining equivalent survival outcomes compared with open procedures [17]. Our early results are consistent with these findings. Despite treating patients with higher BMIs and more advanced disease, features characteristic of Western GC cohorts, we observed low morbidity rates, satisfactory lymph node harvests, and rapid recovery patterns.

One of the most critical considerations in the introduction of LG is the learning curve. Evidence suggests that surgeons require between 50 and 90 distal gastrectomy cases to achieve stable proficiency. A landmark series of 362 cases identified a three-phase learning curve, with performance stabilizing after approximately 60 to 90 procedures [23]. A 2023 meta-regression assessing more than 5,500 laparoscopic distal gastrectomies estimated a learning plateau at 47 cases [24]. For TG, a systematic review of 12 studies found that minimally invasive TG is safe but requires completion of an identifiable learning curve to achieve reproducible oncologic outcomes [25]. Likewise, a Western hand-assisted laparoscopic TG series reported that approximately 44 cases were needed to achieve technical competence and optimize operative time [26].

Our mean operative duration of roughly four hours reflects the expected early-phase learning curve rather than inefficiencies. As our team standardizes operative steps, optimizes intraoperative workflow, and accumulates experience, operative times will predictably decrease. The minimally invasive approach offers clear benefits, including reduced postoperative complications, reduced blood loss, smaller incisions, decreased postoperative pain, earlier mobilization, and shorter hospital stays, although at the expense of longer operative times [27-31]. Early return to oral intake and fewer wound complications are particularly advantageous for older patients with multiple comorbidities, who benefit from reduced surgical stress and a lower incidence of wound complications [32]. Our median length of stay of six days compares favorably with the typical hospital stay of eight to 12 days reported for OG in Greece. This is consistent with a meta-analysis of Western studies including 22,946 patients, showing that LG reduces hospital stay by approximately 2.3 days compared with open surgery [17].

A persistent concern with the minimally invasive approach remains its oncologic adequacy, particularly for advanced GC requiring D2 lymphadenectomy or TG. Recent high-quality Western data strongly support the oncologic validity of the laparoscopic approach. A national cohort from Sweden including 622 patients with cT2-4a disease demonstrated that LG was associated with lower 90-day mortality, higher lymph node yields, and improved overall survival compared with open surgery [33]. Similarly, a 2022 meta-analysis found that laparoscopic TG with D2 lymphadenectomy was comparable to open surgery in terms of oncologic radicality and postoperative outcomes [34]. Taken together, these findings suggest that minimally invasive gastrectomy achieves oncologic standards equivalent to those of open surgery even in high-complexity cases, which is consistent with our own R0 resection rate and adequate lymphadenectomy.

Nevertheless, randomized controlled trials with long-term follow-up remain essential. Recent high-level evidence supports the oncologic safety of laparoscopic distal gastrectomy for locally advanced GC. The multicenter CLASS-01 randomized clinical trial demonstrated that laparoscopic distal gastrectomy with D2 lymphadenectomy produced five-year overall survival rates comparable to OG (72.6% vs. 76.3%, with no statistically significant difference), confirming noninferiority in long-term oncologic outcomes across tumor stages when performed by experienced surgeons in specialized centers [14]. Similarly, the KLASS-02 randomized clinical trial reported five-year overall survival (88.9% vs. 88.7%) and relapse-free survival rates (79.5% vs. 81.1%) that did not differ significantly between laparoscopic and open approaches, with laparoscopic surgery additionally associated with a lower incidence of late complications [35]. Meta-analyses combining individual patient data from KLASS-02 and CLASS-01 reinforce these findings, showing similar five-year overall and recurrence-free survival between laparoscopic and open surgery in patients with locally advanced disease [36].

Our mean lymph node yield of 16 is consistent with recommended thresholds (≥15 nodes) for accurate staging and prognostication. Negative margins were achieved in all cases. While our short follow-up precludes firm conclusions about survival, the absence of local recurrence to date is encouraging. The mean operative time of four hours in our series reflects both the complexity of the procedure and the learning curve inherent in adopting this technique. As our program matures, we expect operative times to decrease while maintaining safety. Performing a totally laparoscopic gastrectomy requires advanced skills in dissection, intracorporeal suturing, and three-dimensional spatial orientation. Academic centers are uniquely positioned to implement this approach, as they can integrate structured training programs, proctorship, and continuous quality monitoring into routine surgical practice. Beyond reporting perioperative and oncologic outcomes, our early experience highlights several practical considerations regarding the implementation of totally laparoscopic gastrectomy.

Adequate expertise in advanced laparoscopy, careful patient selection during the initial phase, and a multidisciplinary approach were important factors facilitating the adoption of this technique. Furthermore, the need for conversion in selected cases emphasizes the importance of maintaining a low threshold for ensuring patient safety during the initial phase. Rigorous adherence to standardized steps and close collaboration among surgical team members were critical components for achieving favorable outcomes. Targeted investment in specialized equipment, such as high-definition laparoscopes and energy devices, also facilitated the transition.

Implementation in the Greek context

Many Greek hospitals face challenges such as limited resources, inconsistent access to advanced equipment, and variations in surgeon training. By demonstrating the feasibility of this technique in an academic setting, our program may serve as a model for other institutions. Key elements for successful implementation include structured training and mentorship for surgeons and residents; a gradual introduction of the technique, starting with lower-risk cases; institutional support for necessary equipment and operating room time; and integration with multidisciplinary oncology care. Additionally, the educational mission of academic hospitals ensures that residents and fellows gain exposure to state-of-the-art techniques, ultimately enhancing the quality of care across the country.

Our study has several limitations. First, the small sample size (14 patients) limits the statistical robustness of our findings and restricts the generalizability of the results to broader patient populations. Therefore, the outcomes reported in this cohort should be interpreted as reflective of our early institutional experience rather than definitive evidence of the overall performance of totally laparoscopic gastrectomy. Second, the retrospective design may introduce selection bias and the potential for incomplete data collection, which may influence the assessment of perioperative and oncologic outcomes. Third, the relatively short follow-up period limits the ability to draw meaningful conclusions regarding long-term survival, recurrence patterns, and functional outcomes, including nutritional status. Similar limitations have been noted in Western series and meta-analyses, emphasizing the need for long-term data to fully validate laparoscopic oncologic outcomes in Western populations [17]. Finally, we did not include a contemporaneous control group undergoing OG at our institution, which would allow more robust comparative analysis within our institution. Future research should address these elements through prospective multicenter studies with larger cohorts and standardized outcome measures.

Our early findings support the continued use and expansion of LG at our hospital. We plan to increase the number of cases by extending the eligibility criteria to include more complex tumors as our experience grows. Additionally, we aim to collect long-term data on patient survival, recurrence rates, and quality of life. We will also conduct cost-effectiveness analyses comparing laparoscopic and open approaches within the Greek healthcare context. Furthermore, we intend to collaborate with other European centers to develop training curricula and registries for minimally invasive gastric surgery. These initiatives will help us determine whether the advantages observed in short-term outcomes translate into durable benefits for patients and the healthcare system.

## Conclusions

LG represents a viable and safe option for the surgical treatment of GC in an academic Greek hospital. In our initial series of 14 patients, the procedure was associated with minimal blood loss, acceptable operative times, a median hospital stay of six days, low complication rates, and adequate oncologic resection outcomes. Although these findings are encouraging, they should be interpreted with caution due to the small sample size and limited follow-up. As laparoscopic techniques continue to evolve and the supporting evidence grows, academic hospitals can play a crucial role in training surgeons, evaluating outcomes, and shaping national guidelines. Ultimately, prospective cohort studies and long-term follow-up are needed to confirm the oncologic equivalence of laparoscopic and open gastrectomy in the Greek population and to establish best practices for patient selection and perioperative care. Our experience demonstrates that, with appropriate preparation and institutional support, the transition to minimally invasive gastric surgery is not only achievable but also has the potential to provide significant benefits to patients, even in healthcare systems with limited resources.
